# Unravelling the genetic architecture of soybean tofu quality traits

**DOI:** 10.1007/s11032-024-01529-x

**Published:** 2025-01-03

**Authors:** Cleo A. Döttinger, Kim A. Steige, Volker Hahn, Kristina Bachteler, Willmar L. Leiser, Xintian Zhu, Tobias Würschum

**Affiliations:** 1 Institute of Plant Breeding, Seed Science and Population Genetics, University of Hohenheim, 70599 Stuttgart, Germany; 2 State Plant Breeding Institute, University of Hohenheim, 70599 Stuttgart, Germany; 3 Taifun-Tofu GmbH, 79108 Freiburg, Germany

**Keywords:** Soybean, Tofu traits, QTL mapping, Genomic selection, Plant breeding

## Abstract

Tofu is a popular soybean (*Glycine max* (L.) Merr.) food with a long tradition in Asia and rising popularity worldwide, including Central Europe. Due to the labour-intensive phenotyping procedures, breeding for improved tofu quality is challenging. Therefore, our objective was to unravel the genetic architecture of traits relevant for tofu production in order to assess the potential of marker-assisted selection and genomic selection in breeding for these traits. To this end, we performed QTL mapping with 188 genotypes from a biparental mapping population. The population was evaluated in a two-location field trial, and tofu was produced in the laboratory to evaluate tofu quality. We identified QTL for all investigated agronomic and quality traits, each explaining between 6.40% and 27.55% of the genotypic variation, including the most important tofu quality traits, tofu yield and tofu hardness. Both traits showed a strong negative correlation (*r* = -0.65), and consequently a pleiotropic QTL on chromosome 10 was found with opposite effects on tofu hardness and tofu weight, highlighting the need to balance selection for both traits. Four QTL identified for tofu hardness jointly explained 68.7% of the genotypic variation and are possible targets for QTL stacking by marker-assisted selection. To exploit also small-effect QTL, genomic selection revealed moderate to high mean prediction accuracies for all traits, ranging from 0.47 to 0.78. In conclusion, inheritance of tofu quality traits is highly quantitative, and both marker-assisted selection and genomic selection present valuable tools to advance tofu quality by soybean breeding.

**Supplementary Information:**

The online version contains supplementary material available at 10.1007/s11032-024-01529-x.

## Introduction

Soybean (*Glycine max* (L.) Merr.) is a leguminous crop of high economic importance. It is primarily used as animal feed, however soybean is also widely used in human nutrition. Soybean products for human consumption include, for example, soybean oil, soy sauce, tempeh, and tofu, i.e. soybean curd. Soybean products are valued for their high protein content and the various health benefits associated with the consumption of soy foods (e.g., Zhang et al. 2003; Lima et al. 2017). With the rise in popularity of plant-based products high in protein, often used as meat alternatives, the demand for tofu and tofu products continues to increase (Statista 2020). Consequently, breeding soybean varieties with improved tofu quality is becoming increasingly important. Amongst other parameters, tofu quality is characterized by tofu hardness, protein content, amino acid profile, and taste (Kurasch et al. 2018a; Wang et al. 2020), making tofu quality evaluation complex. Especially for tofu hardness, the desired levels depend heavily on the intended use and the region of consumption. For example in Asia, a wide variety of tofu texture is offered, whereas the majority of tofu sold in German supermarkets tends to be very firm.

The procedure of evaluating tofu quality is laborious and time consuming, as tofu has to be produced for direct testing, which additionally requires adequate sample sizes of soybeans for destructive measurements. Therefore, selection on a large scale or in the early stages of the breeding cycle is not feasible. In addition to the effect of the genotype, tofu quality traits are influenced by the soybean growing environment (Aziadekey et al. 2002; Poysa and Woodrow 2002; Min et al. 2005; Kurasch et al. 2018a), seed storage conditions after harvest (Kong et al. 2008) and tofu production method (Kurasch et al. 2018a; Huang et al. 2022). Research on associations between tofu quality and soybean agronomic and seed quality traits is inconclusive, making indirect selection, for example by breeding for high protein content, unreliable. Genomics based methods like marker-assisted selection and genomic selection would therefore be valuable tools to drive soybean breeding for superior suitability for tofu production. However, only few QTL mapping studies have been reported. Kurasch et al. (2018b) performed QTL mapping of a range of tofu quality traits, but in each of the three populations identified at maximum one QTL per trait, whereas other studies only focused on mapping loci related to tofu and soymilk yield (e.g., Wang et al. 2008; Huang et al. 2023). Thus, little is known so far about the genetic control of tofu quality traits besides the quantitative inheritance of the different tofu yield and quality parameters (Kurasch et al. 2018b).

A prerequisite for marker-assisted selection is the availability of markers that are diagnostic for or at least closely linked to genetic loci associated with the trait of interest. This is feasible for monogenic traits and for quantitative traits for which individual quantitative trait loci (QTL) explain a large proportion of the genotypic variation of the trait. When, in contrast, many QTL of small effect are responsible for the expression of a complex, highly quantitative trait, marker-assisted selection is not feasible. An alternative approach to assist selection of such traits in breeding is genomic selection. This approach typically utilises a large number of markers covering the whole genome in order to calculate a genomic estimated breeding value that is then used to make selection decisions.

In Germany, production of soybean is on the rise (Eurostat 2024). Furthermore, tofu manufacturers have an interest in locally grown soybeans of high tofu quality. The adaptation to the Central European climate in combination with high tofu quality requires cultivar improvement by plant breeding. To advance soybean breeding with respect to tofu quality, the aims of this study were to investigate the genetic architecture of tofu quality traits by QTL mapping and based on these findings draw conclusions for the potential of marker-assisted and genomic selection as tools to improve tofu quality parameters in breeding.

## Materials and methods

### Plant material and assessment of agronomic traits

One hundred and ninety-eight recombinant inbred lines (RIL) in F4:6 generation (self-pollinated to F4 and then bulked until F6) from a biparental population were tested together with their parental lines, resulting in 200 genotypes in total. Both parental lines were breeding lines high in protein and suitable for tofu production. The field trials were conducted in 2021 in Eckartsweier (EWE) and Hohenheim (HOH) in Southwest Germany. The trials were designed as an alpha lattice design with two replications per location and 20 blocks of 10 genotypes per replication. The soybeans were grown in two-row micro plots of 1.1 m length and 0.32 m row spacing, resulting in a plot size of 0.352 m^2^. Before sowing, the soybean seeds were inoculated with Liquifix and Legumefix (Legume Technology LTD, East Bridgeford, UK). While measuring seed yield was not meaningful due to the small plot size, thousand-seed weight was measured as a yield parameter. Protein and oil content were estimated in the laboratory on seeds dried at 9–10% seed moisture content using near-infrared spectroscopy using a PSS-HOP spectrometer (Polytec GmbH, Waldenbronn, Germany).

### Genotyping

For genotyping, the 198 RILs and their two parental lines were grown in 96-well seedling trays with 3.5 × 3.5 cm wells filled with a 2:1 mix of soil (Substrat 5, Klasmann-Deilmann GmbH, Geeste, Germany) and sand in a growth chamber (12 h light and 12 h darkness; 28°C). Young leaf material was harvested after 15 days and all samples were dried in silica gel for one week.

About 15 mg of dried leaf material was used for DNA extractions following a slightly modified plate-based protocol described by Fazekas et al. (2012). Final washing steps were done using first washing buffer 2 and then 70% ethanol. DNA quantity was measured using the QuantiFluor® dsDNA System kit (Promega, Madison, USA) and all samples were normalised to 10 ng/µl. Genotyping-by-Sequencing (GBS) libraries were constructed following a slightly modified protocol described by Elshire et al. (2011). In short, single i7 indexes were added to allow multiplexing of the libraries. Additionally, an ExoSAP digest using the GenUP™ Exo SAP Kit (biotechrabbit, Berlin, Germany) was added according to the manufacturer’s protocol to remove single stranded DNA and small dimers after the PCR. PCR products were then selected by size using the ProNex® Size-Selective Purification System kit (Promega, Madison, USA). The final quality of the libraries was assessed using the HS NGS Fragment Kit (1–6000 bp, Agilent, Santa Clara, USA). Libraries were sequenced on an Illumina Nova Seq machine (Macrogene and Azenta/Genewiz) generating 150 bp paired-end reads with an average of 4,280,863 reads per genotype (Table S1).

For bioinformatic processing of the GBS data, the GB-eaSy pipeline (Wickland et al. 2017) was followed. First, reads were demultiplexed based on the barcode sequences using the *GBSX tool* (Herten et al. 2015). Quality checks on the raw data were performed using *FastQC* and all further steps were done excluding the second read due to quality issues. Reads were cleaned using the program *fastp* (Chen et al. 2018) and reads were then mapped against the *Wm82.a2.v1* soybean reference genome (Schmutz et al. 2010) using *bwa mem* (Li and Durbin 2009) with default settings. SNP calling was done by first generating the genotype likelihoods using *bcftools mpileup* (Li 2011) using the flags *–skip-indels*, *–min-MQ 20*, and *–min-BQ 20* and then performing the SNP calling on the *mpileup* files using *bcftools call* (Li 2011) with the flags *–multiallelic-caller* and *–variants-only* by chromosome. Finally, SNP files were concatenated using *bcftools concat* (Li 2011). Raw SNP files were filtered with *vcftools* (Danecek et al. 2011) using the *–max-alleles 2*, *–minDP 8* and *–max-missing 0.8* to flags to retain a final SNP file with a total of 315,385 SNPs.

### Preparation of test tofu and assessment of tofu quality traits

In order to assess tofu quality traits, test tofu was prepared using an adaptation of the bench scale method developed by Kurasch et al. (2018a). Seed moisture content of samples of 80 g were measured using a FarmPro moisture analyser (Horn GmbH, Bad Saulgau, Germany). The sample weights were then adjusted to a moisture content of 12% (corrected dry bean weight). Afterwards, the samples were soaked in tap water for 24 h at a temperature of about 8 °C. To prepare soymilk, the drained soybeans were ground with 480 ml of tap water for one minute at 10,000 rpm. The slurry was then filtered with a filter hose to separate the raw soymilk from the pulp. The soymilk was heated to 98.5 °C for 3 min with constant stirring at 150 rpm. An estimate of solid contents dissolved in the soymilk in °Brix was obtained with a PR-32α refractometer (ATAGO Co., LTD, Tokyo, Japan). A measured value was accepted once the value was constant for three consecutive measurement runs. The soymilk was then adjusted to 8.4°Brix by adding heated tap water. Of the adjusted soymilk, 500 ml were used to further process into tofu. As a coagulant, 0.45 g CaSO_4_ was dissolved in 20.55 ml of a 2.95% Nigari solution (MgCl_2_ 6H_2_O) prepared with tap water. For that, the coagulant was added to the soymilk at a temperature between 84.5 and 85.5 °C. The mixture was stirred for six seconds at 250 rpm and then placed into a water bath at 84.5–85.5 °C for 20 min. Afterwards, the curd was broken up using a curd breaker and left in the water bath. After 10 min, the whey was drained from the curd with a sieve for 45 s. The curd was filled into moulds of 7.5 cm diameter and pressed with rising pressure from 0.1 to 1 to 2 to 3 to 4 Pa for 4 min each. The pressed tofu was stored in cold water at around 8 °C for 12 to 18 h overnight. After draining, the tofu was weighed to obtain tofu weight (TW).

For additional tofu quality parameters, the soaking factor (SF) was calculated as .

$SF=\frac{soaked\;bean\;weight}{corrected\;dry\;bean\;weight}$. Tofu hardness (TH) in N was obtained using a texturometer (Zwick AG, Ulm, Germany). Soymilk weight (SMW) in g refers to the weight of the soymilk adjusted to 8.4°Brix. Tofu yield (TY) was calculated as $TY=\frac{TW}{corrected\;dry\;bean\;weight}\times\frac{SMW}{500}$. The tofu value (TV) was calculated as $TV= {TY}^{2}\times TH$. The number of hard beans was counted in each sample. Soybeans that did not take up water during the soaking process were considered as hard beans.

### Phenotypic analysis

Best linear unbiased estimators (BLUE), best linear unbiased predictors (BLUP) and variance components were calculated for all traits using the model

$${y}_{ijkm}=\mu +{g}_{i}+{l}_{j}+{gl}_{ij}+{r}_{jk}+{b}_{jkm}+{\varepsilon }_{ijkm},$$

where *y*_*ijkm*_ represents the phenotypic observation, *g*_*i*_ the i^th^ genotype, *l*_*j*_ the j^th^ test location, *gl*_*ij*_ is the genotype-by-location interaction, *r*_*jk*_ is the replication *k* in location *j*, *b*_*jkm*_ is the m^th^ block in replication *k* and location *j*, and *ε*_*ijkm*_ is the corresponding error term. To calculate BLUE values, *g*_*i*_ was considered as a fixed effect. To calculate BLUP values and variance components, *g*_*i*_ was considered a random effect. The counts of hard beans were transformed by square root transformation prior to the estimation of BLUEs, BLUPs and variance components in order to fulfil the model assumptions. Thirteen small negative BLUE values were set to zero for biological plausibility. Heritability (*h*^*2*^) was calculated as proposed by Cullis et al. (2006) and described by Piepho and Möhring (2007) as follows:

$$\overline{{h }^{2}}=1-\overline{\frac{{\vartheta }_{BLUP}}{2{\sigma }_{g}^{2}}},$$

where $\overline{{\vartheta }_{BLUP}}$ describes the mean variance of the difference between two BLUP values and ${\sigma }_{g}^{2}$ describes the genotypic variance.

All analyses were conducted using the programming language *R* (R Core Team 2021) in the *RStudio* environment (Posit team 2023). BLUEs, BLUPs and variance components were estimated using the *R* package *ASReml-R* (Butler 2021).

### Linkage map construction and QTL analysis

A linkage map was constructed for the QTL mapping using the *R* package *ASMap* (Taylor and Butler 2017). Prior to map construction, missing marker values were imputed and the marker data was corrected for miscalled alleles using the *R* package *ABHgenotypeR* (Reuscher and Furuta 2016). In addition, genotypes with heterozygosity above 30% and markers with excessive segregation distortion were removed. During linkage map construction, markers were additionally filtered for inflated double crossovers. Physical and genetic marker orders were plotted against each other and markers showing a strong deviation from the expected pattern were removed. After these filtering steps, the final linkage map was constructed with the remaining 188 genotypes and 2,452 SNP markers. The same data set was then also used for QTL mapping.

QTL analysis was conducted using a composite interval mapping (CIM) approach with an additive genetic model and the software *PlabMQTL* (Utz 2011). Empirical logarithm of odds (LOD) thresholds with a genome-wide significance level of $\alpha \le 0.10$ were computed for each trait with permutation tests of 1000 runs (Churchill and Doerge 1994). The modified Bayesian information criterion (mBIC) was used for cofactor selection. The proportion of genotypic variance explained by each QTL (*p*_*G*_) was calculated as the value of ${p}_{G}=\frac{{R}_{part}^{2}}{{h}^{2}}$ normalised according to Zhu et al. (2004), where ${R}_{part}^{2}$ describes the proportion of the phenotypic variance explained by the respective QTL. QTL results were validated by 200 independent fivefold cross-validations (Utz et al. 2000).

### Genomic prediction

Genomic prediction was conducted for all traits by ridge regression BLUP (RR-BLUP) using the *rrBLUP* package in *R* (Endelman 2011). The genotypes and SNPs from the linkage map were used and missing values were imputed using the software *LinkImpute* (Money et al. 2015). Using BLUEs across locations, 1000 runs of a fivefold cross-validation were applied, where the training set (TS) included a random set of 80% of the genotypes and the prediction set (PS) contained the remaining 20%. The prediction accuracy was calculated as ${r}_{GP}=\frac{{r}_{MP}}{h}$, where *r*_*MP*_ represents the correlation between genomic estimated breeding values (GEBV) and observed BLUE values and *h* represents the square root of the heritability (Lande and Thompson 1990; Dekkers 2007).

## Results

Heritabilities for thousand-seed weight, protein content and oil content were medium to high at 0.65, 0.72 and 0.62, respectively (Table 1). For the tofu quality traits, heritabilities were generally lower ranging from 0.23 for tofu weight to 0.66 for the number of hard beans. The genotypic variance was highly significant (*P* < 0.001) for all traits except for tofu weight. The effect of the trial location was highly significant (*P* < 0.001) as well, for all traits except for the soaking factor. In contrast, the genotype-by-location interaction variance was significant only for thousand-seed weight, oil content and hard beans.

**Table 1:** Summary statistics of the evaluated traits. Mean of BLUE values ($\overline{\text{X} }$), genotypic variance (${\upsigma }_{\text{G}}^{2}$), location variance (${\upsigma }_{\text{L}}^{2}$), the respective interaction variance (${\upsigma }_{\text{GL}}^{2}$), error variance (${\upsigma }_{\upvarepsilon }^{2}$), and heritability (${\text{h}}^{2}$) for thousand-seed weight in gram (TSW), protein content in percent (PC), oil content in percent (OC), soaking factor (SF), soymilk weight in gram (SM), tofu weight in gram (TW), tofu yield (TY), tofu hardness in Newton (TH), tofu value (TV), and the square root of counted hard beans (HB)

Trait	$\overline{X }$^+^	${\sigma }_{G}^{2}$	${\sigma }_{L}^{2}$	${\sigma }_{GL}^{2}$	${\sigma }_{\varepsilon }^{2}$	${h}^{2}$
TSW	227.97	102.29***	66.78***	44.09***	123.44	0.65
PC	46.73	0.63***	0.11***	9.33e^−8ns^	0.92	0.72
OC	15.91	0.10***	0.34***	0.04**	0.16	0.62
SF	2.11	5.33e^−4^***	0.05e^−4ns^	0.78e^−4ns^	9.86e^−4^	0.64
HB	1.05	0.32***	0.11***	0.09**	0.46	0.66
SM	571.55	61.82***	76.47***	6.73^ns^	146.93	0.59
TW	139.29	5.08^ns^	18.39***	2.98^ns^	59.34	0.23
TY	1.97	2.55e^−3^***	4.07e^−3^***	0.47e^−3ns^	10.00e^−3^	0.47
TH	78.96	33.05***	62.06***	1.04e^−5ns^	196.42	0.39
TV	300.95	212.00***	101.83***	1.87e^−4ns^	1848.20	0.30

*P < 0.05, **P < 0.01, ***P < 0.001, ^ns^not significant

^+^Mean values for HB were transformed back to represent the count of hard beans

**Fig. 1:**
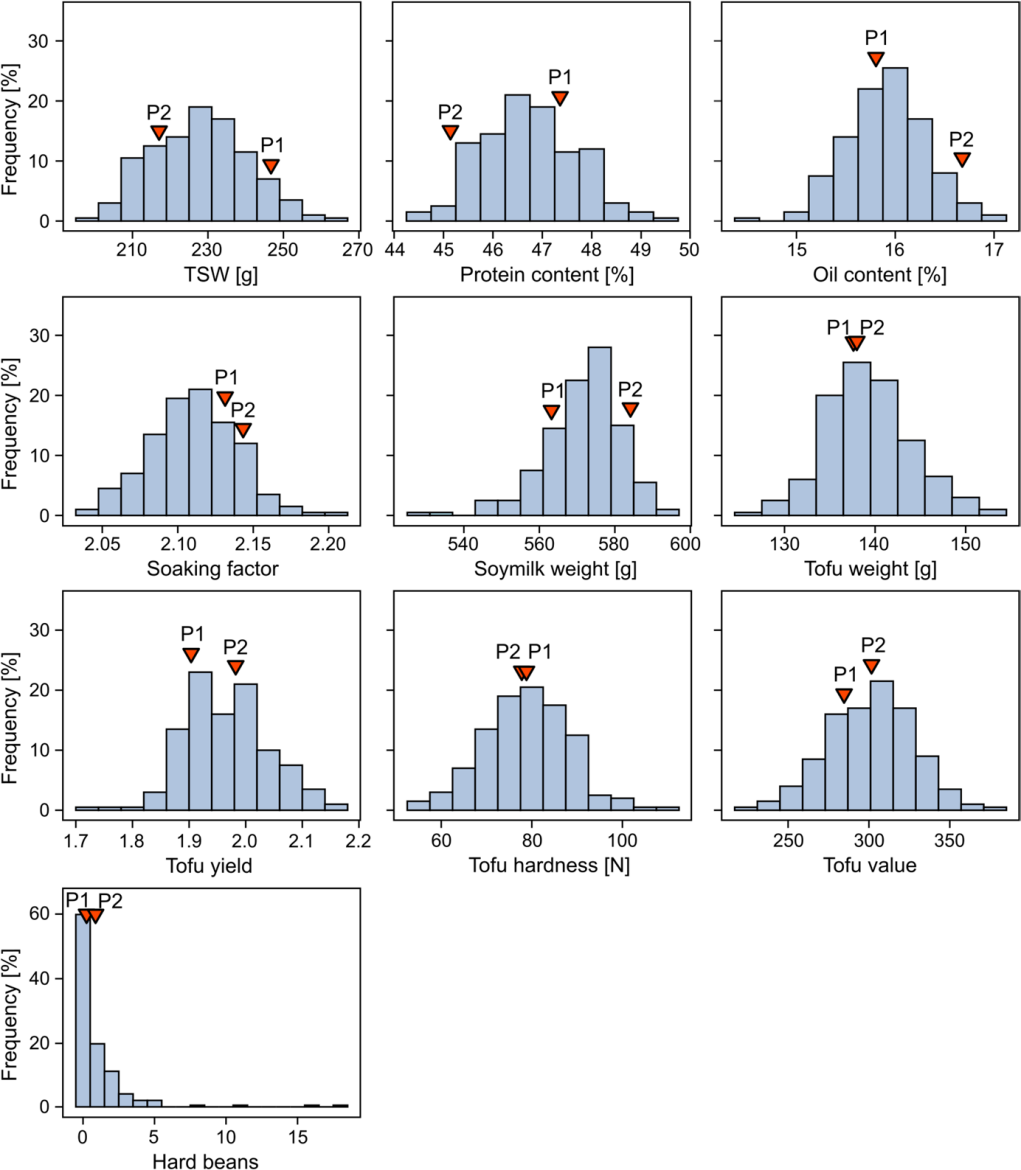
Trait distributions. Distribution of BLUE values across locations for the traits thousand-seed weight (TSW), protein content, oil content, soaking factor, soymilk weight, tofu weight, tofu yield, tofu hardness, tofu value, and number of hard beans. The performance of the parental genotypes is indicated by red triangles. Note, BLUE values for hard bean counts were estimated based on square root transformed values, the resulting BLUEs were transformed back for this figure

BLUEs across locations varied substantially between the genotypes and approximately followed a normal distribution for all traits except for hard bean count (Fig. 1). On average, the genotypes showed high thousand-seed weight (227.97 g), very high protein content (46.73%) and lower oil content (15.91%) (Table 1). For tofu quality traits, tofu hardness ranged from 54.67 N to 108.11 N and the mean of 78.96 N is slightly above the producer’s threshold of 75 N. In contrast, ranging between 1.74 and 2.16, mean tofu yield of 1.97 lay slightly below the producer’s threshold of 2.00. The two parental genotypes showed very similar performance for all tofu quality traits except for the soymilk weight (Fig. 1). For thousand-seed weight, protein content and oil content, the parents differed more strongly, as parent 1 showed higher thousand-seed weight and protein content, whereas parent 2 was higher in oil content.

**Fig. 2:**
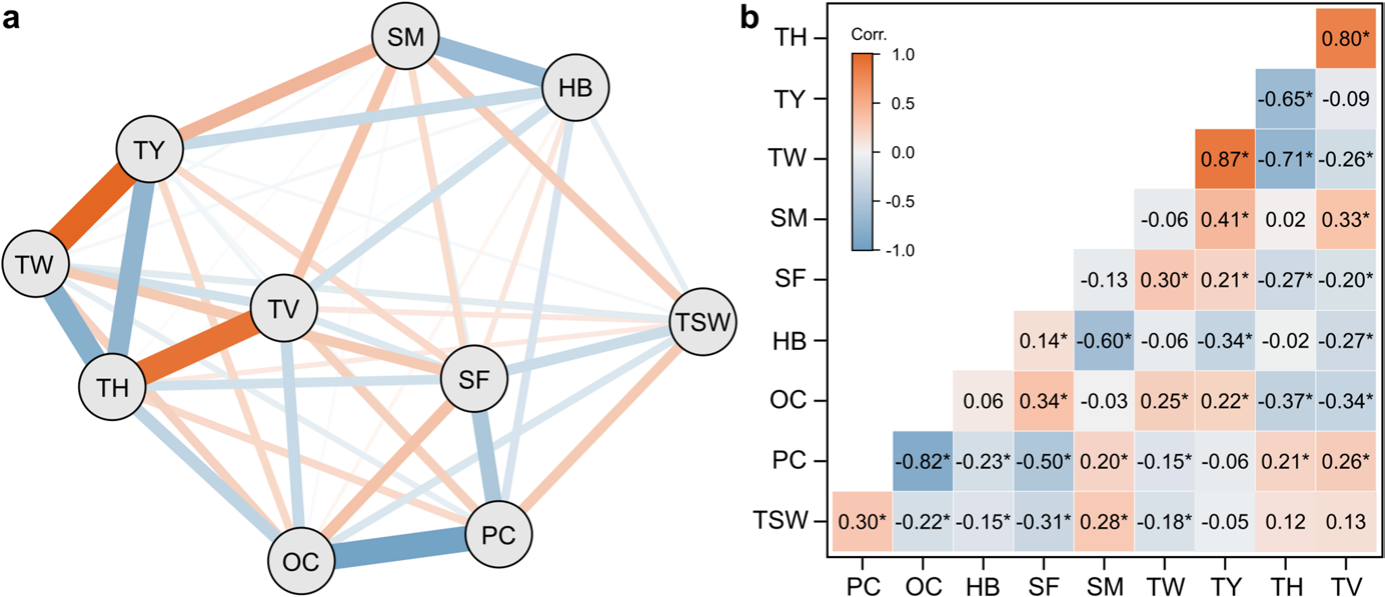
Correlation among the investigated traits. a Network plot and b matrix with the correlations among the traits thousand-seed weight (TSW), protein content (PC), oil content (OC), soaking factor (SF), soymilk weight (SM), tofu weight (TW), tofu yield (TY), tofu hardness (TH), tofu value (TV), and number of hard beans (HB). Asterisk indicates correlation coefficients significantly different from zero at the 5% significance level

The yield parameter thousand-seed weight was significantly (*P* < 0.05) positively correlated with protein content and soymilk weight at a moderate level, whereas it was negatively correlated with oil content, tofu weight, hard bean count and soaking factor (Fig. 2). However, thousand-seed weight showed no significant correlation with tofu yield, tofu hardness and tofu value. Protein content was weakly to moderately positively correlated with soymilk weight, tofu value and tofu hardness and negatively correlated with tofu weight, soaking factor and count of hard beans. Tofu yield showed a strong positive correlation with tofu weight (*r* = 0.87) and moderate positive correlation with soymilk weight. In contrast, tofu yield was moderately negatively correlated with hard bean count (*r* = −0.34) and tofu hardness (*r* = −0.65). In addition, tofu hardness was strongly positively correlated with the tofu value (*r* = 0.80), whereas the correlation with tofu weight was strongly negative (*r* = −0.71). Tofu value was further moderately positively correlated with soymilk weight and negatively correlated with tofu weight and hard bean count. Hard bean count was also negatively correlated with soymilk weight at *r* = −0.60.

QTL mapping identified at least one QTL for all traits (Tab. 2, Fig. S1). For thousand-seed weight, three QTL were found on chromosomes 7, 14 and 16, explaining between 9.41% and 12.33% of the genotypic variance. For protein content, we found five QTL on chromosomes 5, 11, 12, 14 and 16 explaining 6.40–13.96% of the genotypic variance and three different QTL were detected for oil content explaining 9.43–16.17%. On chromosome 4, a pleiotropic QTL was found for the number of hard beans and soymilk, explaining 13.59% and 16.15% of the genotypic variance, respectively, for which the favourable allele was associated with a reduction in hard beans of 0.06 and an increase of 3.49 g in soymilk weight. Another pleiotropic QTL was found on chromosome 10 for tofu weight, explaining 27.55% of genotypic variance, tofu hardness (20.65%) and tofu value (20.18%). For tofu weight, the favourable allele was contributed by parent 1, leading to an increase of 1.39 g in tofu weight, whereas tofu hardness and tofu value were increased by 3.44 N and 7.59, respectively, by the allele from parent 2. Additional major QTL that explained over 20% of the respective genotypic variance were found on chromosome 13 for tofu yield and on chromosome 5 for tofu value, where the allele of parent 2 was associated with an increase of 0.03 and 8.84, respectively. Overall, for the tofu quality traits, parent 2 contributed the majority of the favourable alleles. The major QTL showed substantial effects on the respective traits across, as well as within locations (Fig. S2).

**Table 2:** Results from QTL mapping. Chromosome (Chr.) and position (Pos) of detected QTL and their support interval (SI) in centiMorgan (cM), flanking markers indicating their physical position, logarithm of odds (LOD), donor of the favourable allele, the additive effect (Add. eff.), proportion of genotypic variance explained by the QTL (*p*_*G*_), and the frequency in which the QTL was observed in 200 independent five-fold cross-validation runs (Freq. CV), shown for the traits thousand-seed weight (TSW), protein content (PC), oil content (OC), soaking factor (SF), count of hard beans (HB), soymilk weight (SM), tofu weight (TW), tofu yield (TY), tofu hardness (TH), and tofu value (TV)

Trait	Chr.	Pos. (SI) (cM)	Left marker	Right marker	LOD	Donor (fav. allele)	Add. eff.^+^	*p*_*G*_ (%)	Freq. CV
TSW	7	57 (53–61)	Chr07_5770755	Chr07_6463334	3.15	Parent 1	3.34	9.41	0.26
	14	61 (53–66)	Chr14_8232119	Chr14_9109810	3.59	Parent 1	3.54	10.67	0.46
	16	117 (106–130)	Chr16_28461478	Chr16_35670141	4.18	Parent 1	5.20	12.33	0.68
PC	5	131 (128–133)	Chr05_41123747	Chr05_41392595	2.88	Parent 2	0.22	6.40	0.16
	11	73 (58–79)	Chr11_25910388	Chr11_18186161	4.51	Parent 1	0.29	9.81	0.57
	12	145 (142–146)	Chr12_35603615	Chr12_35692321	6.57	Parent 1	0.35	13.96	0.42
	14	9 (5–24)	Chr14_1159327	Chr14_1437584	4.13	Parent 2	0.26	9.04	0.25
	16	144 (134–146)	Chr16_36955239	Chr16_37051444	5.26	Parent 1	0.30	11.36	0.33
OC	9	0 (0–2)	Chr09_223061	Chr09_305244	6.44	Parent 2	0.15	16.17	0.79
	12	187 (186–187)	Chr12_38815575	Chr12_39085077	4.70	Parent 2	0.13	12.05	0.26
	18	96 (75–104)	Chr18_10268740	Chr18_12826385	3.63	Parent 2	0.11	9.43	0.19
SF	10	129 (120–139)	Chr10_43599219	Chr10_48347975	3.41	Parent 1	0.01	9.69	0.37
	17	121 (118–122)	Chr17_40412430	Chr17_40584687	4.17	Parent 1	0.01	11.72	0.70
	18	105 (103–107)	Chr18_16111507	Chr18_16686711	6.98	Parent 2	0.01	19.01	0.76
HB	4	59 (55–60)	Chr04_7990022	Chr04_8395715	4.74	Parent 2	−0.06	13.59	0.53
	14	24 (21–26)	Chr14_3546556	Chr14_3734218	3.04	Parent 2	−0.04	8.92	0.26
SM	2	120 (118–126)	Chr02_38862876	Chr02_39191621	2.69	Parent 2	2.55	9.53	0.12
	4	59 (58–65)	Chr04_7990022	Chr04_8395715	4.66	Parent 2	3.49	16.15	0.58
	4	122 (114–134)	Chr04_46792598	Chr04_48009592	3.02	Parent 2	3.10	10.69	0.40
TW	10	19 (17–22)	Chr10_2920019	Chr10_3139957	3.09	Parent 1	1.39	27.55	0.21
TY	13	120 (118–122)	Chr13_30645621	Chr13_30673726	4.54	Parent 2	0.03	20.39	0.40
TH	1	76 (72–84)	Chr01_47186726	Chr01_48805860	4.10	Parent 2	2.86	15.74	0.22
	8	28 (24–32)	Chr08_4651791	Chr08_6045309	3.69	Parent 2	2.70	14.24	0.28
	10	14 (11–18)	Chr10_2348299	Chr10_2906572	5.47	Parent 2	3.44	20.65	0.52
	18	47 (34–61)	Chr18_2098093	Chr18_3883865	4.75	Parent 1	3.35	18.10	0.20
TV	5	1 (0–3)	Chr05_1420686	Chr05_1545718	4.35	Parent 2	8.84	26.21	0.72
	8	152 (137–156)	Chr08_44206039	Chr08_45163890	2.69	Parent 2	6.93	16.55	0.25
	10	16 (11–19)	Chr10_2348299	Chr10_2906572	3.31	Parent 2	7.59	20.18	0.40

^+^For QTL mapping HB counts transformed with square root transformation were used, here the values were transformed back to represent actual HB counts

**Fig. 3:**
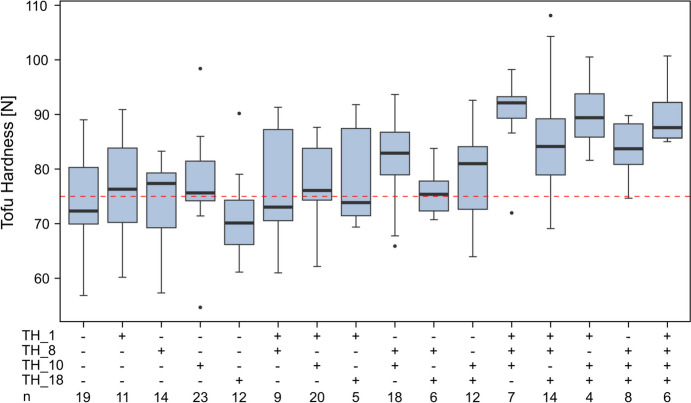
The effect of QTL stacking. The boxplot illustrates the effect of combining favourable alleles of the four identified QTL for tofu hardness on chromosomes 1, 8, 10 and 18 in 188 genotypes. + indicates the presence of the homozygous favourable allele, -indicates the homozygous unfavourable allele, heterozygous loci and missing values. The red dotted line represents the quality threshold of 75 N desired in German tofu production

We exemplarily used the QTL identified for tofu hardness to evaluate the effect of QTL stacking, which revealed a substantial effect on the target trait as several allelic combinations of at least three favourable alleles consistently performed above the desired threshold of 75 N (Fig. 3).

**Fig. 4:**
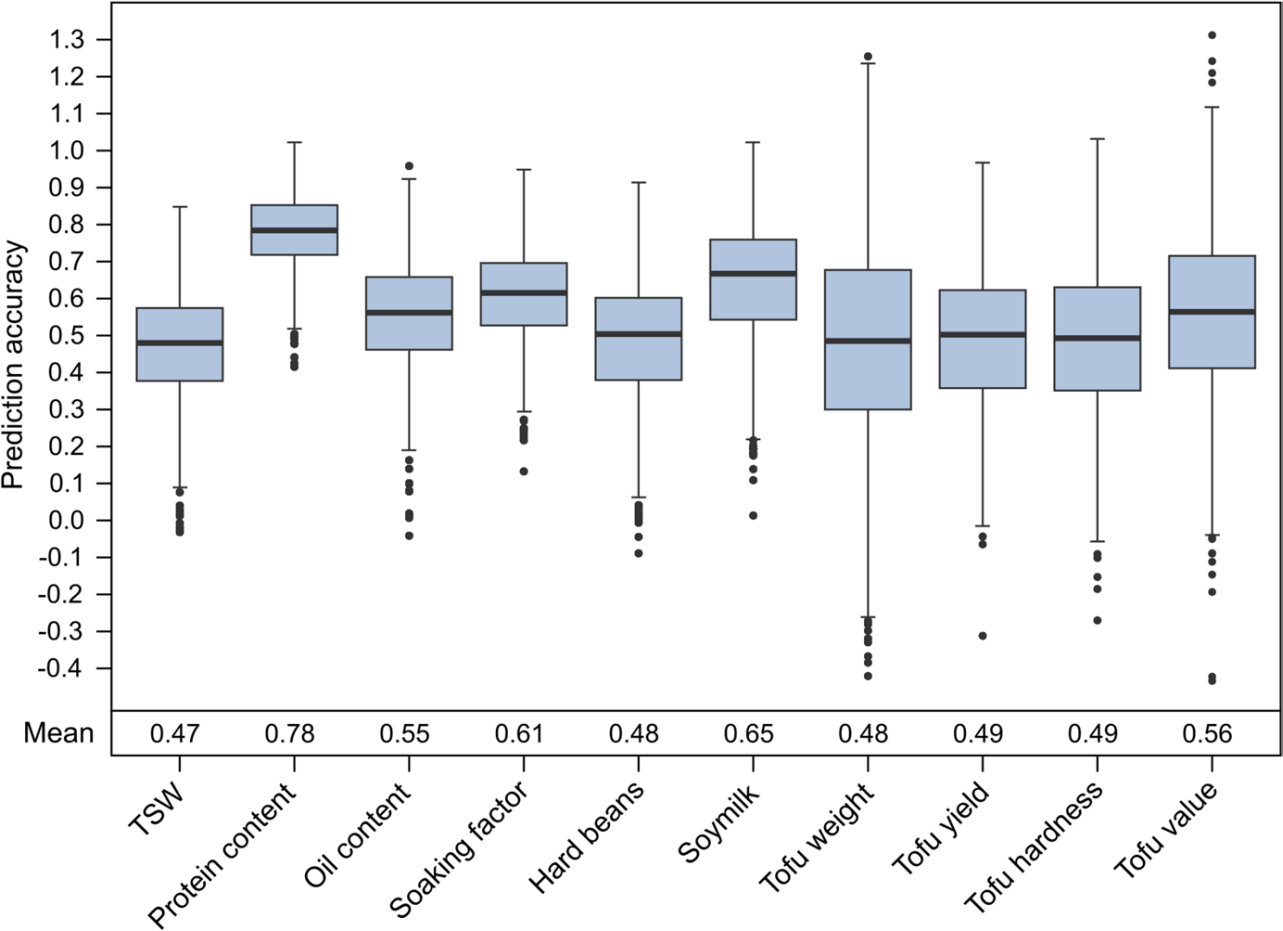
The potential of genomic prediction. Genomic prediction accuracies from 1000 runs of cross-validation and their respective means are shown

Genomic prediction with the same 188 genotypes and the 2,452 genome-wide markers used for QTL mapping revealed moderate mean prediction accuracies for all traits, ranging from 0.45 for thousand-seed weight to 0.61 for protein content (Fig. 4). Five-fold cross-validation also revealed a large variation in prediction accuracy between the runs. For example, prediction accuracies for tofu hardness ranged from −0.20 to 0.76 with a mean of 0.49, which for the majority of the tofu quality traits was in a similar range.

## Discussion

### Breeding for soybean tofu quality

The increasing trend towards vegetarian or vegan nutrition moves traits related to tofu quality to the foreground in soybean breeding programs targeted at human consumption. For the biparental mapping population underlying this study, we specifically used two parental lines that both are suitable for tofu production. The reasoning behind this is that such crosses are typical for applied breeding programs and thus the population allows investigating the variation and its genetic control relevant for tofu quality breeding.

The tofu quality traits showed low to medium heritabilities between 0.23 for tofu value and 0.66 for the number of hard beans (Tab. 1). Notably, both parental lines showed similar performance in most tofu quality traits and consequently, the genotypic variance might be rather small in comparison to the error variance, resulting in a lower heritability (Fig. 1, Tab. 1). This conclusion is supported by the fact that the heritability was especially low for traits for which the parents performed most similarly and showed a relatively high error variance (i.e. tofu weight, tofu yield, tofu hardness and tofu value). The significant effect of both the soybean genotype and the growing environment, specifically on tofu yield and hardness, is in accordance with previous studies (Wang et al. 1983; Mullin et al. 2001; Aziadekey et al. 2002; Min et al. 2005; Kurasch et al. 2018b). A source of the rather large error effects likely lies in inhomogeneities in the test tofu laboratory. When establishing the bench-scale tofu testing method followed in this study, Kurasch et al. (2018a) found strong effects of the team preparing the test tofu as well as the day of tofu production. Consequently, several steps of the procedure were subsequently standardized or even automated. Nevertheless, the various steps of the tofu production process in combination with the small amount of seeds used for it, are still a source for confounding effects. However, despite the medium heritabilities found in this study, our results highlight the feasibility of breeding soybean with improved tofu quality.

The genotypes of the mapping population varied substantially for the two most important tofu quality traits, tofu yield (between 1.74 and 2.16) and especially for tofu hardness (ranging from 54.67 to 108.11 N) (Fig. 1). For tofu yield, the tofu manufacturer desires a minimum value of 2.0 with the optimum of 2.2. Many genotypes therefore showed too low tofu yield for production purposes, including the parental lines not reaching the cut-off level of 2.0. For tofu hardness, the desired range lies between 75 and 120 N. In addition to the rather low tofu yields, many genotypes also showed values below this desired minimum value. This may be due to the environment, which, as shown here, also has a substantial effect on these two traits. However, while the absolute trait level is relevant for tofu production, the present variation of the traits is the decisive factor for successful QTL mapping. Interestingly, we observed transgressive segregation for all traits, meaning that there were progeny with trait values outperforming the superior parent or lower than the inferior parent. This means that both parents carry favourable and unfavourable alleles for the target traits that are newly combined in the progeny. Consequently, the combination of favourable alleles from both parents results in genotypes with a performance superior to either parent, which are candidates for new varieties and illustrate how breeding progress for tofu quality can be achieved.

The relationship between agronomic traits and tofu quality is unclear. In our mapping population, no correlation was found between thousand-seed weight and tofu hardness or tofu yield. For thousand-seed weight and protein content or soymilk weight, however, moderate positive correlations of 0.30 and 0.28, respectively, could be observed. Regarding protein content, the literature on its influence on tofu quality is conflicting. Previously, a positive association between seed protein content and tofu yield has been reported (Bhardwaj et al. 1999). In other studies, however, this conclusion could not be corroborated (Wang et al. 1983; Lim et al. 1990; Kurasch et al. 2018a, 2018b). Non-significant or low correlation coefficients between protein content and tofu yield parameters and tofu hardness were found in our study (Fig. 2), supporting the conclusion that the protein content by itself has a low relevance and it is more likely the protein composition that determines tofu quality. Notably, however, this does not rule out an effect of protein content, for which a certain minimum may be required as a basis for tofu quality. In our soybean material, all genotypes had a high protein content between 44.61% and 49.25% and thus, in populations including variation in the lower protein content range, the effect of protein content on tofu quality traits might be larger. The analysis of trait correlations also revealed the well-known strong negative correlation between protein and oil content, explaining why, if present, they often have opposite effects on tofu traits.

The tofu quality traits showed substantial positive as well as negative correlations among each other. For example, a strong negative correlation between tofu yield and tofu hardness (*r* = −0.65; Fig. 2) was observed, that has also been reported in previous studies (Min et al. 2005; Kurasch et al. 2018a). Therefore, a simultaneous improvement of both traits is challenging since tofu yield is closely linked to higher water content, whereas firmer tofu tends to hold less water (Min et al. 2005). Consequently, a balance between both traits needs to be achieved to produce tofu with a texture suited to consumer preferences while providing an adequate tofu yield. Taken together, also in the cross of two tofu-type soybeans, there is substantial variation in the resulting progeny and the mapping population is consequently well suited to dissect the genetic architecture of tofu quality traits.

### The genetic architecture of tofu quality traits

Characterization of the genetic architecture of target traits holds the potential to identify major QTL that could then be utilized in marker-assisted selection. In our study, we identified QTL for all evaluated traits, including the tofu quality traits.

We identified three QTL for thousand-seed weight on chromosomes 7, 14 and 16, for which the favourable allele was always contributed by parent 1, the parent with the higher seed weight. Several QTL have been reported for seed weight on chromosome 7 (Orf et al. 1999; Csanádi et al. 2001; Hoeck et al. 2003; Teng et al. 2009; Li et al. 2010; Yan et al. 2014), of which one overlaps with our QTL (Teng et al. 2009). Seed weight QTL have also been reported on chromosome 14 (Specht et al. 2001; Hoeck et al. 2003; Liu et al. 2011) and chromosome 16 (Maughan et al. 1996; Kim et al. 2010).

For the number of hard beans, which significantly impairs soymilk weight, tofu yield and tofu value (Fig. 2), we identified two QTL on chromosomes 4 and 14, where the allele from parent 2 was associated with fewer hard beans in both cases. For traits related to seed coat permeability, several QTL have been reported (e.g., Liu et al. 2007; Ren et al. 2024), but none on chromosome 4 or 14. However, Kurasch et al. (2018b) reported a QTL on chromosome 4 for the weight gain after 25 h of soaking that overlaps with the QTL identified here. Hard beans are characterised by not taking up water during the soaking process. Therefore, both findings indicate causal genetic variation in that region of chromosome 4. In our study, this QTL was pleiotropic for soymilk weight. Since hard beans cannot be processed into soymilk and are removed before blending the seeds, this QTL likely indirectly influences soymilk yield by affecting the soaking behaviour of the seeds.

There was no substantial correlation between protein content and tofu traits in our population and none of the QTL for protein content overlapped with those of the tofu traits. However, two QTL on chromosome 8 associated with tofu value in our population have also been reported for soluble protein content (Lu et al. 2013), indicating at least some association between (soluble) protein and tofu quality. Moreover, a QTL for tofu hardness has been identified in a similar region of chromosome 8 (Kurasch et al. 2018b). This supports a possible role of water-soluble protein in the expression of tofu quality traits (Lu et al. 2013). Further publications suggest that tofu yield parameters and hardness are linked to protein composition rather than protein content alone. Especially the ratio of the storage proteins glycinin (11S) to β-conglycinin (7S) may affect tofu quality, with a higher proportion of 11S protein being associated with firmer tofu in the majority of publications (e.g., Saio et al. 1971; Yagasaki et al. 2000; Renkema et al. 2001; Poysa et al. 2006; Stanojevic et al. 2011). Notably, the influence of the storage proteins and their subunit composition strongly relies on the tofu production method and the soybean variety, which may explain contrasting findings (Utsumi and Kinsella 1985; Cai and Chang 1999; Yang and James 2023). Interestingly, the gene *Gy4*, encoding the glycinin G4 subunit on chromosome 10 (SoyBase, www.soybase.org), is located within the confidence interval of the QTL we detected for tofu weight, which is pleiotropic for tofu hardness and tofu value (Table 2, Fig. S1). In the genotyping-by-sequencing data, the gene region was not covered. We therefore employed re-sequencing data of the parental lines (Zhu et al. 2023) to search for polymorphisms in *Gy4*. This revealed no polymorphisms in the *Gy4* coding sequence, the introns, the promoter or the downstream region. This indicates that if *Gy4* is the causal gene underlying this QTL, this may be due to expression differences caused by sequence polymorphisms farther away from the gene, or that despite being a very plausible candidate, the QTL is not caused by *Gy4* but another gene in this chromosomal region, which requires further research on the molecular level. In addition, two of the QTL found for tofu hardness in this study overlap with previously reported protein content QTL on chromosome 1 (Pandurangan et al. 2012; Mao et al. 2013) and on chromosome 18 (Jun et al. 2008; Lu et al. 2013; Mao et al. 2013), indicating a possible influence of protein content on tofu hardness despite the observed relatively low phenotypic correlation.

Taken together, the tofu quality traits can be considered as complex traits but with some medium- to large-effect QTL being present. In addition, the substantial effects of the identified breeding-relevant major QTL on the respective traits within single locations indicate their effectiveness across various environments (Fig. S2). As the tofu traits are laborious to phenotype and in addition are strongly influenced by the growing environment and other non-genetic factors, being able to directly address and exploit such QTL, and with them larger proportions of the genotypic variance, would be a big step forward in targeted breeding for tofu quality.

### Potential of marker-assisted and genomic selection to advance tofu quality breeding

Marker-assisted selection is a valuable tool to directly exploit causal genetic loci in advancing a trait of interest. However, when QTL explain only a small proportion of genotypic variance, this approach is not suitable to drive the improvement of the targeted traits. Together, the four QTL identified in our study for tofu hardness could explain 68.7% of the genotypic variance (Table 2). We therefore exemplarily used this trait to explore the potential of QTL stacking, i.e. the combination of several favourable QTL alleles in a genotype. This revealed that the combination of at least three QTL leads to tofu hardness values consistently above the manufacturer’s desired threshold of 75 N for most combinations (Fig. 3). The major QTL on chromosome 10 that shows the strongest effect on ensuring meeting this threshold, however, was pleiotropic for tofu weight and tofu value. In line with the negative correlation of tofu weight with the other two traits, the allele from parent 2 was found to increase tofu hardness and tofu value, but had a negative effect on tofu weight. In general, a pleiotropic QTL can be due to one gene affecting two or more traits or can also be caused by closely linked loci, each affecting only one trait. In the latter case, the causal alleles can be separated by recombination, even though in practice this may require substantial efforts. In case of a single locus with pleiotropic effect, a breeder must decide which allele to select for and consequently which trait to increase and which to decrease. The decrease could be better accepted for the trait for which a compensation through other loci is more easily achieved. Nonetheless, the result on QTL stacking indicates that a marker-assisted selection approach might be feasible to efficiently exploit major QTL and with them a substantial proportion of the genotypic variance. Notably, however, QTL always depend on the studied population and results are often not transferable to other populations. The loci need to segregate within the population and their effects might vary in different genetic backgrounds. In a similar study, Kurasch et al. (2018b) found a QTL for tofu hardness on chromosome 8 in only one of three evaluated biparental populations. Interestingly, the pleiotropic QTL they identified for tofu hardness and tofu value at 62 and 64 cM on chromosome 8, respectively, did not overlap with the tofu hardness QTL identified in our population on chromosome 8 at 28 cM. This further highlights the strong dependency of the QTL results on the studied population, which limits the transferability required for marker-assisted selection. Therefore, in order to make use of the identified QTL, their genomic location needs to be fine-mapped and their effect on the trait of interest needs to be verified in different populations.

A complementary approach to marker-assisted selection is genomic selection, that offers the ability to utilise genomic information for the improvement of highly quantitative traits. In this study, mean prediction accuracies were moderate to high, ranging between 0.47 for thousand-seed weight and 0.78 for protein content (Fig. 4). Given the mentioned complexity in phenotyping the tofu quality traits, this can be very valuable in breeding. However, the variation of prediction accuracies between the cross-validation runs was rather large for all traits. For example, prediction accuracies for tofu hardness ranged from −0.27 to 1.03 with a mean of 0.49. A variation this large indicates a strong dependency of the respective prediction accuracy on the composition of the training and prediction sets. In a similar study, evaluating a very similar set of tofu quality parameters in two RIL populations, Kurasch et al. (2018b) found in part substantially lower mean prediction accuracies for tofu weight, tofu yield, tofu hardness and tofu value but with a similarly large variation. Thus, the accuracy of genomic prediction of tofu quality traits needs to be increased further and made more reliable for application in practical soybean breeding. This can likely be achieved by larger training sets including different genetic material representative of the breeding program. Ideally, the phenotypic assessment of the traits can also be improved further, so that the heritability increases and the data underlying the training of the prediction model become more robust. A potential limitation of genomic selection are the associated costs, as the approach requires genome-wide marker data. Taken together, marker-assisted selection and genomic selection show potential to assist breeding of tofu quality traits, but both require further validation and optimization before they can be routinely employed in breeding programs.

## Conclusion and outlook

Tofu quality traits are becoming increasingly important in soybean breeding programs targeted at the food market. In this study, we evaluated a biparental population and observed substantial variation and transgressive segregation for all investigated traits, illustrating that an improvement of tofu quality traits can also be achieved in best-by-best crosses that are typical in applied breeding programs. We also observed intricate interrelations among the tofu traits, including negative correlations, which need to be balanced during selection, potentially using index selection. QTL mapping revealed some moderate- to large-effect QTL for all tofu quality traits, but the results also illustrate an in general complex inheritance with many additional small-effect QTL. Consequently, both marker-assisted and genomic selection hold potential for breeding, or even their joint use or integration, but both require further research and optimization. In conclusion, soybean breeding for improved tofu quality traits is feasible to meet the increasing demand and diversity of vegetarian and vegan products.

## Supplementary Information

Below is the link to the electronic supplementary material.

Supplementary file1 (PDF 632 KB)

Supplementary file2 (XLSX 22 KB)

## Data Availability

Data is contained in the supplementary material.
