# Supplementary material for: Unravelling the genetic architecture of soybean tofu quality traits
Source: Mol Breed. 2025 Jan 3;45(1):8. doi: 10.1007/s11032-024-01529-x (PMC11699088; doi:10.1007/s11032-024-01529-x)
Supplement: Supplementary file 1 — Supplementary file1 (PDF 632 KB) [file 11032_2024_1529_MOESM1_ESM.pdf]

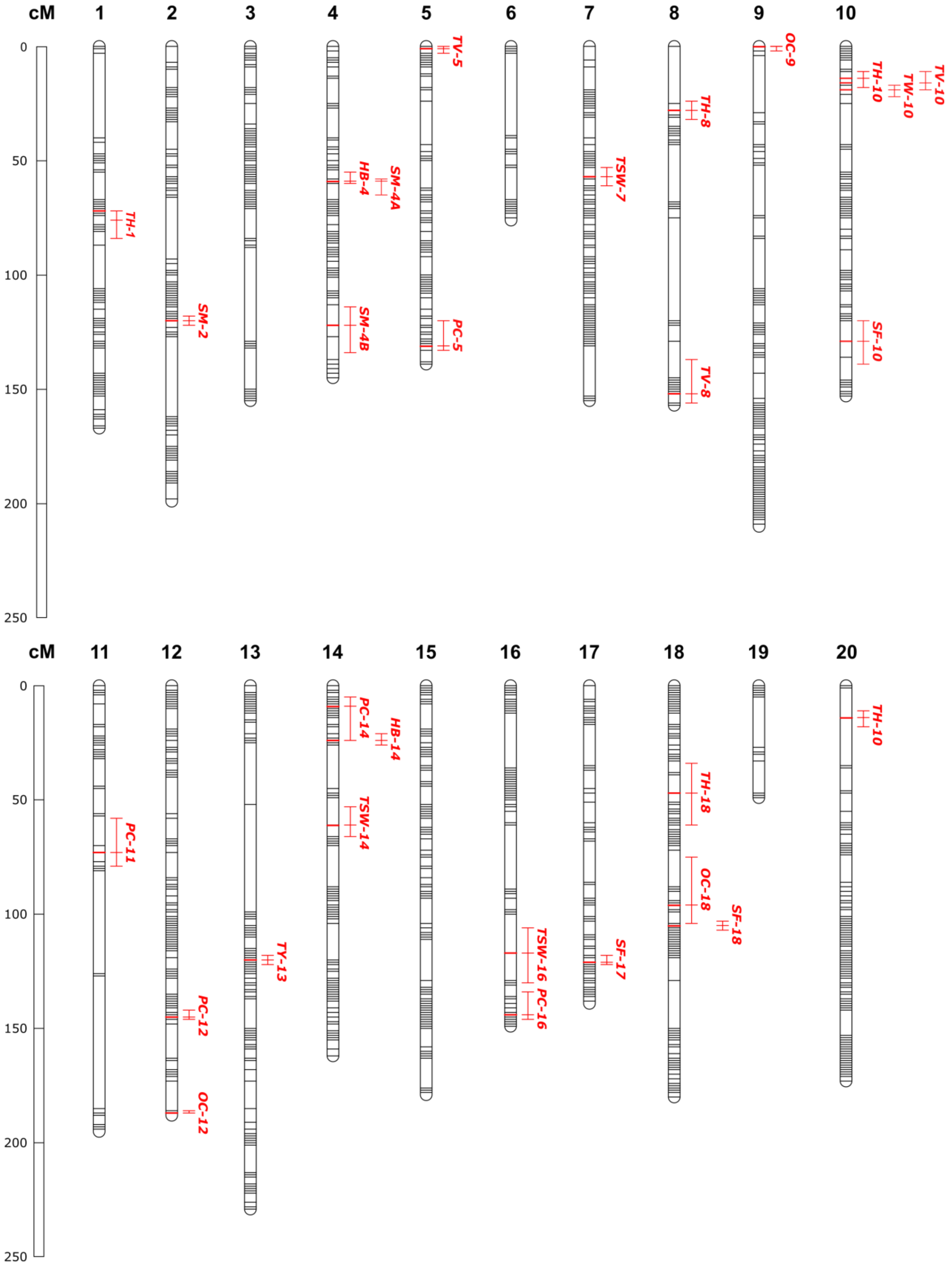

Fig. S1 Linkage map of the 20 chromosomes of soybean, including the QTL identified for the investigated traits. Black horizontal lines indicate the positions of the markers used for QTL mapping. The positions of the QTL are shown in red, with the supporting interval given in whisker plots. The QTL are named according to the trait as thousand-seed weight (TSW), protein content (PC), oil content (OC), soaking factor (SF), count of hard beans (HB), soymilk weight (SM), tofu weight (TW), tofu yield (TY), tofu hardness (TH), and tofu value (TV), followed by the number of the chromosome.

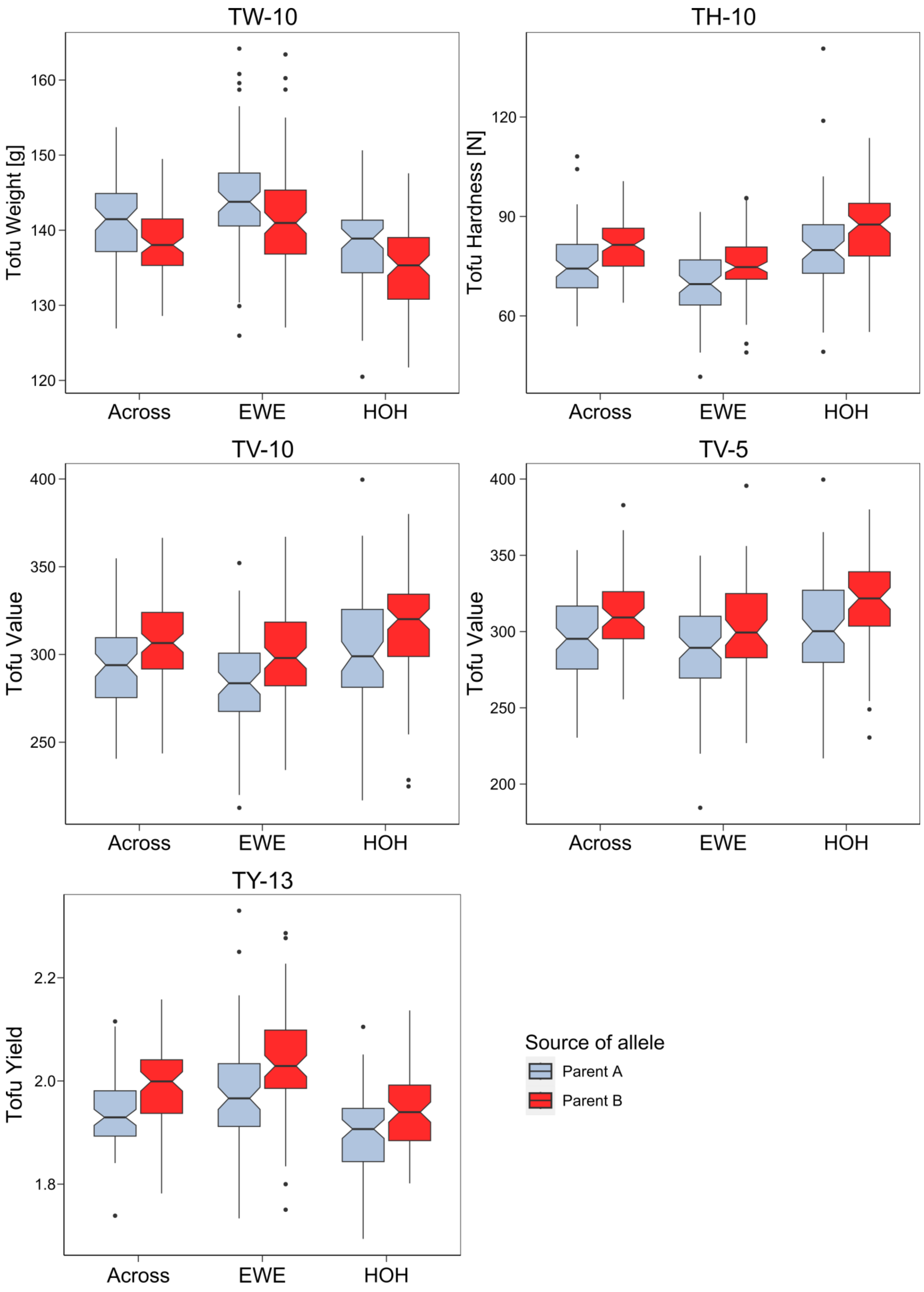

Fig. S2 Boxplots of allele performance of major QTL at single locations. Effects of the alleles of major, breeding-relevant QTL identified across locations and explaining more than 20 % of the genotypic variance shown for the BLUEs across locations and for the two locations EWE and HOH. QTL are on chromosome 10 for tofu weight (TW-10), tofu hardness (TH-10) and tofu value (TV-10), for tofu value on chromosome 5 (TV-5), and for tofu yield on chromosome 13 (TY-13).
